# Participation in Physical, Social, and Religious Activity and Risk of Depression in the Elderly: A Community-Based Three-Year Longitudinal Study in Korea

**DOI:** 10.1371/journal.pone.0132838

**Published:** 2015-07-14

**Authors:** Hyun Woong Roh, Chang Hyung Hong, Yunhwan Lee, Byoung Hoon Oh, Kang Soo Lee, Ki Jung Chang, Dae Ryong Kang, Jinhee Kim, SooJin Lee, Joung Hwan Back, Young Ki Chung, Ki Young Lim, Jai Sung Noh, Dongsoo Kim, Sang Joon Son

**Affiliations:** 1 Department of Psychiatry, Ajou University School of Medicine, Suwon, Republic of Korea; 2 Institute on Aging, Ajou University Medical Center, Suwon, Republic of Korea; 3 Department of Preventive Medicine and Public Health, Ajou University School of Medicine, Suwon, Republic of Korea; 4 Department of Psychiatry and Institute of Behavioral Science in Medicine, Yonsei University College of Medicine, Seoul, Republic of Korea; 5 Department of Psychiatry, CHA University School of Medicine, CHA Hospital, Gangnam, Republic of Korea; 6 Department of Medical Humanities & Social Medicine, Office of Biostatistics, Ajou University School of Medicine, Suwon, Republic of Korea; 7 Department of Medicare Administration, Backseok Arts University, Seoul, Republic Korea; 8 National Health Insurance Service, Seoul, Republic of Korea; McMaster University, CANADA

## Abstract

**Background:**

We examined the longitudinal association between participation in individual or combinations of physical, social, and religious activity and risk of depression in the elderly.

**Methods:**

Elderly subjects aged ≥60 years who completed the Living Profiles of Older People Survey in Korea (n = 6,647) were included. The baseline assessment, Wave 1, was conducted in 2008, and a follow-up assessment, Wave 2, was conducted in 2011. We defined participation in frequent physical activity as ≥3 times weekly (at least 30 minutes per activity). Frequent participation in social and religious activity was defined as ≥1 activity weekly. The primary outcome was depression at 3-year follow up.

**Results:**

Multivariable logistic regression analysis showed that subjects who participated in frequent physical, social, and religious activity had an adjusted odds ratio of 0.81 (95% confidence interval [CI], 0.69–0.96), 0.87 (95% CI, 0.75–1.00), and 0.78 (95% CI, 0.67–0.90), respectively, compared with participants who did not participate in each activity. Participants who participated in only one type of activity frequently and participants who participated in two or three types of activities frequently had an adjusted odds ratio of 0.86 (95% CI, 0.75–0.98) and 0.64 (95% CI, 0.52–0.79), respectively, compared with participants who did not participate in any type of physical, social, and religious activity frequently.

**Conclusion:**

Participation in physical, social, and religious activity was associated with decreased risk of depression in the elderly. In addition, risk of depression was much lower in the elderly people who participated in two or three of the above-mentioned types of activity than that in the elderly who did not.

## Introduction

Depression in the elderly is a public health concern attracting worldwide attention. Ongoing research is focused on relevant biological, psychosocial, and environmental risk factors. In the past few decades, intensive efforts have been made to identify psychosocial risk factors for depression that allow modification and intervention [1–4]. While medication and various types of psychotherapy are still the primary treatment options for depression in the elderly, lifestyle modification has recently received attention as a safe and low-cost option for augmenting the management of depression [5]. Among various lifestyle factors, participation in physical and social activity has attracted much research attention. Researchers have found relatively consistent and strong evidence for the efficacy of these activities in improving mood [6–8]. In addition, owing to the current emphasis on spirituality, participation in religious activity has recently attracted research attention from psychiatrists and geriatricians as a factor responsible for increasing resilience or improving mood [9]. In the present study, we investigated the association between these three promising lifestyle factors and the risk of depression in the elderly through a community-based 3-year longitudinal study in Korea.

Participation in physical activity was the first subject of our interest. A recent systematic review suggested that various types of physical activity, including elements of endurance and strength training, have a protective effect in the elderly against depression severity [10]. These activities usually include a moderate- to high-intensity exercise program, at a frequency of 3 to 5 times weekly, 30 to 50 minutes per session, for 3 to 4 months. The protective effects of physical activity involve various neurobiological mechanisms, including increased neural plasticity and neurochemical change, as well as some psychosocial mechanisms, including self-efficacy and a sense of mastery [11–14].

Participation in social activity is the second subject of our interest. According to the World Health Organization, social activity is an important component of “Active aging” [15]. Indeed, preceding research has suggested that participation in social activity, such as a neighborhood association, retired or elderly association or charitable association, alleviates depressive symptoms [16,17]. In addition, a recent systematic review of interventions for social isolation suggests that social activity in a group format was more likely to be beneficial compared to a one-to-one format [18]. The protective effects of social activity involve various psychosocial mechanisms, including increased social support and buffering of distress [17,19–21].

Participation in religious activity is the third subject of our interest. This subject has recently been attracting more attention. A few studies have suggested that participating in religious activity on a regular basis is a protective factor for older adults’ mental health and protects against common mental disorders, including depression. [9,22–26]. However, some of these studies were based on a cross-sectional design, from which it was difficult to draw conclusions regarding causality [9,24,26]. A few studies based on a longitudinal design included only limited subjects, such as patients who were ill, or had insufficient numbers of subjects [22,23,27]. The protective effect of religious activity involves neurobiological and psychosocial mechanisms, including increased cortical thickness and resilience [28–30]. Considering the aforementioned promising protective effects of physical, social and religious activity participation, there is clearly a need for a large-scale longitudinal study involving the elderly in local communities. Additionally, the subject of the necessity for a study pursuing concrete evidence for the correlation or combination effect of these activities, rather than simple presumption, has continuously been raised [31].

The main objective of the present study was to assess the longitudinal association between physical, social and religious activity participation and the risk of depression in the elderly. We investigated not only the effect of each type of activity, but also combinatorial effects. In contrast to preceding studies that focused mostly on the functional or qualitative aspects of a single risk factor, we quantitatively evaluated the three risk factors in combination [21,32–34]. A balance between these two approaches would provide the theoretical foundation for development of an intervention in the area of community mental health service, to induce behavioral changes in elderly [35].

## Methods

### Study sample

The Institutional Review Board at Keimyung University, which administered the survey, approved the study protocol. At baseline, participants were informed of the survey and invited to participate via telephone. Approximately 4 weeks later, trained interviewers from Hankook Research visited participants’ houses and obtained written informed consent, and the full survey and questionnaire.

Participants responded to the Living Profiles of Older People Survey (LPOPS), conducted by the Ministry of Health Welfare and Family in Korea. The LPOPS was planned as a nationwide 3-year interval longitudinal survey, and was initiated in 2008. We applied the geographic and demographic information based on a two-stage stratified cluster sampling method. First, the data were stratified by seven constituent metropolitan areas and nine constituent provinces that were further stratified by urban (neighborhood) and rural area (town or township). The participants were then divided into to the 25 secondary geographic strata. The geographic stratification covered all 16 metropolitan areas or provinces in Korea. Sampling within secondary geographic strata employed auxiliary stratification indices, such as gender ratio and average age obtained from surveys to yield a representative sample.

The baseline assessment, Wave 1, was conducted in 2008, and a follow-up assessment, Wave 2, was conducted in 2011. Of the 19,007 non-institutionalized elderly aged ≥60 years who were invited to LPOPS, 15,146 completed the baseline assessment. The response rate of LPOPS at the baseline assessment was 79.7%. At the 3 year follow-up of the baseline participants, 902 (6.0%) of the elderly patients had died and 4,241 (28.0%) had not completed the survey due to refusal, hospitalization, institutionalization or loss of contact. This resulted in a final follow-up sample of 10,003 (66.6%). A more detailed description of the LPOPS is available elsewhere [36,37].

**Fig 1:**
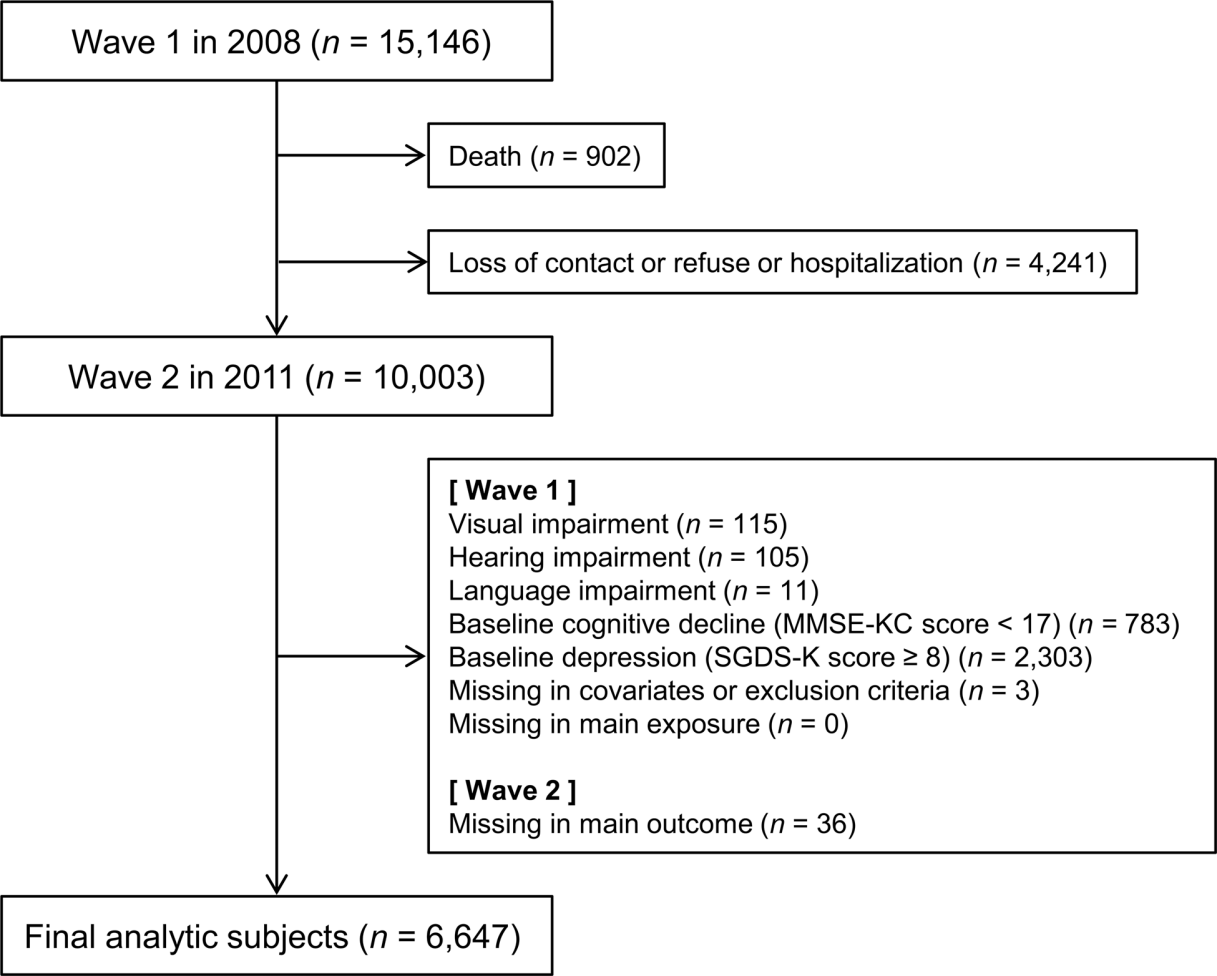
Flow chart of participants.

We included participants who completed both Waves and excluded those who met the following criteria: visual impairment, hearing impairment, language impairment, cognitive impairment and depression, at baseline. The exclusion process was done to remove the effect of other conditions which could have a negative influence on engagement in the activities under study. A total of 6,647 participants were included in the final analysis (Fig 1).

MMSE-KC: Korean version of the Mini Mental State Examination, SGDS-K: Korean version of the 15-item Geriatric Depression Scale.

### Measures

#### Depression

Depression was the primary outcome of interest in our study. It was measured using the Korean version of the 15-item Geriatric Depression Scale (SGDS-K), which was introduced by Sheik and Yesavage and translated into Korean by Bae and Cho [38,39]. SGDS-K scores range from 0 to 15 and are highly correlated with the 30-item Korean version of the Geriatric Depression Scale. A community-based study in Korea suggested that the optimal cut-off point for screening major depressive disorder was a SGDS-K score of 8 or higher, and the sensitivity and specificity of the findings was 93.6% and 76.0%, respectively. The SGDS-K had satisfactory reliability (Cronbach’s alpha of 0.90) and validity [40].

#### Physical activity participation

Above-moderate physical activity participation was one of our primary interests, and it was assessed with verbal questions posed by trained interviewers. Participants were asked, “How often do you participate in above-moderate physical activity in a week? This includes activities such as power walking, swimming, table tennis, badminton, stretch exercises, dancing, yoga, etc.” Eight responses were available for the first question, ranging from 0 = “no physical activity” to 7 = “daily.” Participants who responded with at least 1 physical activity per week were asked, “For how long do you participate in above-moderate physical activity, at once (by minute)?” Based on existing research and the distribution of responses in our study, we defined frequent above-moderate physical activity participation as ≥3 times a week and at least 30 minutes per activity [11,41]. The percentage of participants who had frequent above-moderate physical activity participation was 19.8%.

#### Social activity participation & religious activity participation

Participation in social activity and religious activity were also among our primary interests, and these were assessed with verbal questions posed by trained interviewers. Participants were asked, “Do you participate in social activity? This includes activities such as senior center, hall for the elderly, fraternities, reunions, clan gatherings, etc.” and “Do you participate in religious activity? This includes activities such as church, catholic church, temple, etc.” Yes or no responses were available for the question. Participants who responded in the affirmative were asked, “How often do you participate in that activity?” Five responses were available for the second question, ranging from 1 = “once or twice a year” to 5 = “equal to or more than 4 times a week.” Based on existing research and the distribution of responses in our study, we defined frequent social or religious activity participation as participation in ≥1 activity per week [31,42,43]. The percentage of subjects with frequent participation in social and religious activity was 26.4% and 25.0%, respectively.

#### Group classification by physical, social and religious activity participation

We subsequently classified participants into three groups based on responses to questions on participation in physical, social, and religious activities. Group A consisted of participants who responded that they did not participate in any type of physical, social or religious activity frequently. Group B consisted of participants who responded that they participated in only one type of activity frequently. Group C consisted of participants who responded that they participated in two or three types of activity frequently. As a result, 2,901 (43.6%) participants were classified into Group A, 2,826 (42.5%) participants were classified into Group B, and 920 (13.8%) participants were classified into Group C (Table 1).

**Table 1:** Group classification by physical, social, and religious activity participation (*n* = 6,647).

Type of activity	*n*	%
**Physical activity participation** ^**a**^		
Less than three times a week	5,328	80.2
Equal to or more than three times a week	1,319	19.8
**Social activity participation**		
Less than once a week	4,892	73.6
Equal to or more than once a week	1,755	26.4
**Religious activity participation**		
Less than once a week	4,983	75.0
Equal to or more than once a week	1,664	25.0
**Group classification by activity participation** ^**a**^		
Group A (No frequent physical, social, religious activity)	2,901	43.6
Group B (Only one type of activity frequently)	2,826	42.5
Group C (Two types or three types of activity frequently)	920	13.8

^a^At least 30 minutes per session of physical activity.

#### Covariates

Covariates assessed in the study were age, gender, education, quartiles of household income, smoking, alcohol intake, number of diseases (diagnosed by physician), disability, cognitive function, and baseline SGDS-K score.

Only diseases that were diagnosed by a physician were included in the number of diseases. Our questionnaire included: four cardiovascular diseases (hypertension, stroke, hyperlipidemia, and angina pectoris), two endocrine diseases (diabetes and thyroid disease), four musculoskeletal diseases (arthritis, osteoporosis, back pain, and sciatica), three pulmonary diseases (chronic obstructive pulmonary disease, asthma, and tuberculosis), three eye and ear diseases (cataracts, glaucoma, and chronic otitis media), oncologic diseases (all cancers), three gastrointestinal diseases (ulcer or gastritis, hepatitis, and liver cirrhosis), four genitourinary diseases (chronic renal failure, benign prostate hyperplasia, urinary incontinence, and sexually transmitted infection), and other diseases (anemia and chronic dermatologic disease).

Disability was measured using the Korean version of the Instrumental Activities of Daily Living scale (K-IADL). The K-IADL includes 10 questions on instrumental daily living, and scores range from 10 to 33. Lower scores indicate a higher capability of instrumental daily living. The K-IADL has satisfactory reliability (Cronbach’s alpha of 0.94) and validity [44,45].

Cognitive function was measured with the Korean version of the Mini Mental State Examination (MMSE-KC). The MMSE-KC includes 26-items for temporal orientation, registration, recollection, concentration, calculation, language, understanding, and judgment. MMSE-KC scores range from 0 to 30. Higher scores indicate a higher level of cognitive function. A community-based study in Korea defined the cut-off point on the MMSE-KC for screening dementia as a score of ≤16, and the sensitivity and specificity of the findings were 76.8% and 90.0%, respectively. The MMSE-KC had satisfactory reliability (Cronbach’s alpha of 0.83) and validity [46,47]. A trained interviewer used a written questionnaire to assess other covariates.

### Statistical analysis

Descriptive statistics to summarize the data and cross-tabulation to identify their distribution were performed. Categorical variables were compared using the Chi-square test for trend, and continuous variables were compared using an ANOVA for each of the groups based on activity participation. Correlation analyses between the three activities were conducted using the phi coefficient. Depression was assessed by SGDS-K score. After checking for collinearity, multiple logistic regression analysis was conducted to examine the association of activity participation and risk of depression (SGDS-K score ≥ 8) in the elderly. We built the first model with all the covariates and the three individual activities (physical, social and religious), and a second model with all the covariates and the group classification variable. The Hosmer and Lemeshow test was used to confirm goodness of fit for the logistic regression model. Unadjusted as well as adjusted odds ratios (ORs) were presented using a 95% confidence interval (CI). After that, multiple linear regression analysis was conducted to examine the association between activity participation and depressive symptoms (SGDS-K score as a continuous variable) in the elderly. A *p*-value < 0.05 was considered statistically significant. SPSS software, version 18.0 (SPSS Inc., Chicago, IL, USA) was used for all analyses.

## Results

### General characteristics of participants

The general characteristics of the participants are provided in Table 2. Of the 6,647 participants without depression at baseline, 1,443 (21.7%) of the participants had developed depression (SGDS-K score ≥ 8) at follow-up. The age range of the total participants was 60 to 95 years, and the mean age of the participants was 69.8 years (SD = 6.1). Approximately 44.5% of the participants were men and 55.5% were women. Among the total participants, 76.5% had some form of formal education. Of these, 42.7% had elementary school level (1–6 years), 27.3% had middle to high school level (7–12 years), and 6.5% had college level (over 13 years) education. Household income was categorized into a quartile variable based on the 15,146 persons in Wave 1. Approximately 14.0% of the participants were current smokers, and 37.6% were current drinkers. The mean number of diseases was 1.8 (SD = 1.5), the mean K-IADL score was 10.4 (SD = 1.5) and the mean MMSE-KC score was 24.9 (SD = 3.2). There were statistically significant baseline group differences in age, gender, education, smoking, number of diseases, K-IADL score, MMSE-KC score, and SGDS-K score (Table 2).

**Table 2:** Baseline characteristics of the study participants by activity participation (n = 6,647).

Variable	Group classification by activity participation ^a^				F or *x* ^2^ ^b^	*p*-value
	Total (n = 6,647)	Group A (n = 2,901)	Group B (n = 2,826)	Group C (n = 920)		
**Age**	69.8 ± 6.1	69.2 ± 6.1	70.2 ± 6.1	69.8 ± 6.1	20.741	<0.001
**Gender**					11.993	0.001
Men	2,960 (44.5)	1,367 (47.1)	1,206 (42.7)	387 (42.1)		
Women	3,687 (55.5)	1,534 (52.9)	1,620 (57.3)	533 (57.9)		
**Education**					12.971	<0.001
No education	1,563 (23.5)	695 (24.0)	685 (24.2)	183 (19.9)		
1–6 years	2,837 (42.7)	1,246 (43.9)	1,199 (42.4)	392 (42.6)		
7–12 years	1,813 (27.3)	807 (27.8)	760 (26.9)	246 (26.7)		
≥13 years	434 (6.5)	153 (5.3)	182 (6.4)	99 (10.0)		
**Quartiles (Q) of household income**					1.119	0.290
Q1 (lowest)	1,208 (18.2)	525 (18.1)	526 (18.6)	157 (17.1)		
Q2	1,711 (25.7)	762 (26.3)	708 (25.1)	241 (26.2)		
Q3	1,885 (28.4)	843 (29.1)	779 (27.6)	263 (28.6)		
Q4 (highest)	1,843 (27.7)	771 (28.6)	813 (28.8)	259 (28.2)		
**Smoking**					17.084	<0.001
Ex or none smoker	5,719 (86.0)	2,446 (84.3)	2,450 (86.7)	823 (89.5)		
Current smoker	928 (14.0)	455 (15.7)	376 (13.3)	97 (10.5)		
**Alcohol intake**					3.381	0.066
No drinking	4,148 (62.4)	1,763 (60.8)	1,794 (63.5)	591 (64.2)		
Once a week (or less)	1,305 (19.6)	618 (21.3)	505 (17.9)	182 (19.8)		
Two times a week (or more)	1,194 (18.0)	520 (17.9)	527 (18.6)	147 (16.0)		
**Number of disease**	1.8 ± 1.5	1.7 ± 1.4	1.8 ± 1.5	1.8 ± 1.5	7.815	<0.001
**K-IADL score**	10.4 ± 1.5	10.4 ± 1.6	10.4 ± 1.5	10.2 ± 0.9	7.832	<0.001
**MMSE-KC score**	24.9 ± 3.2	25.0 ± 3.3	24.9 ± 3.2	25.2 ± 3.2	3.022	0.049
**SGDS-K score**	2.5 ± 2.3	2.7 ± 2.3	2.4 ± 2.2	1.9 ± 2.1	42.432	<0.001

Values are mean ± standard deviation (SD) or *n* (%).

^a^ Group A (participants who did not participate in any type of physical, social, and religious activity frequently), group B (participants who participated in only one type of activity frequently), group C (participants who participated in two or three types of activity frequently).

^b^ Analysis of variance (ANOVA) for continuous variables and *x* ^2^-test for categorical variables.

K-IADL: Korean version of Instrumental Activities of Daily Living, MMSE-KC: Korean version of Mini Mental State Examination, SGDS-K: Korean version of 15-item Geriatric Depression Scale.

### Correlation between physical, social and religious activity participation

Correlation analyses between the three activities were conducted using the phi coefficient. Among them, participation in social activity (r = 0.024, *p* = 0.053) and religious activity (r = -0.011, *p* = 0.349) were not correlated with participation in physical activity. Only participation in social activity (r = -0.054, *p* < 0.001) was weakly correlated with religious activity, but its effect size was not large (Table 3).

**Table 3:** Correlation analysis between physical, social and religious activity participation (n = 6,647).

	Physical activity	Social activity	Religious activity
**Physical activity**	1.000		
**Social activity**	0.024 (0.053)	1.000	
**Religious activity**	-0.011 (0.349)	-0.054 (<0.001)	1.000

Values are phi coefficient (*p*-value).

### Longitudinal association between physical, social, and religious activity participation and risk of depression at follow-up

In the multivariable analysis, age, gender, education, household income, smoking, alcohol intake, number of diseases, K-IADL score, MMSE-KC score and SGDS-K score were regarded as covariates.

Multiple logistic regression analysis was conducted to examine the association of activity participation and risk of depression (SGDS-K score ≥ 8) in the elderly. We built the first model with all the covariates and the three individual activities (physical, social, and religious activity), and a second model with all the covariates and the group classification variable.

In the first model, participants who participated in physical activity ≥3 times weekly (at least 30 minutes per activity) had an adjusted OR of 0.81 (95% CI, 0.69–0.96), compared to participants who did not. Participants who participated in social activity and religious activity ≥1 time per week had adjusted ORs of 0.87 (95% CI, 0.75–1.00) and 0.78 (95% CI, 0.67–0.90), respectively, compared to participants who did not. In the second model, participants who participated in only one type of activity frequently (Group B) and participants who participated in two or three types of activity frequently (Group C) had adjusted ORs of 0.86 (95% CI, 0.75–0.98) and 0.64 (95% CI, 0.52–0.79), respectively, compared to participants who did not participate in any type of physical, social, and religious activity frequently (Group A).

Multiple linear regression analysis was also conducted to examine the association between activity participation and depressive symptoms (SGDS-K score as a continuous variable) in the elderly, using similar approach (Table 4).

**Table 4:** Longitudinal association between physical, social and religious activity participation and risk of depression at follow-up (*n* = 6,647).

Variable	Multiple logistic regression model		Multiple linear regression model	
	Adjusted OR (95% CI)	*p*-value	β (SE)	*p*-value
**Model 1** ^**a**^				
**Physical activity participation** ^**b**^				
Less than three times a week (ref)	ref.		ref.	
Equal to or more than three times a week	0.81 (0.69–0.96)	0.014	-0.42 (0.12)	0.001
**Social activity participation**				
Less than once a week (ref)	ref.		ref.	
Equal to or more than once a week	0.87 (0.75–1.00)	0.047	-0.28 (0.11)	0.011
**Religious activity participation**				
Less than once a week (ref)	ref.		ref.	
Equal to or more than once a week	0.78 (0.67–0.90)	0.001	-0.34 (0.11)	0.003
**Model 2** ^**a**^				
**Group classification by activity participation**				
Group A (No frequent physical, social, and religious activity) (ref)	ref.		ref.	
Group B (Only one type of activity frequently)	0.86 (0.75–0.98)	0.020	-0.41 (0.10)	<0.001
Group C (Two types or three types of activity frequently)	0.64 (0.52–0.79)	<0.001	-0.70 (0.15)	<0.001

^a^ Model 1: All covariates + three individual activities (physical, social and religious), Model 2: All covariates + group classification variables

^b^At least 30 minutes per session of physical activity.

SE: Standard error, SGDS-K: Korean version of 15-item Geriatric Depression Scale.

## Discussion

The results showed that participation in physical, social, and religious activity was associated with decreased risk of depression in the elderly, after adjusting for known confounding factors. The odds of depression decreased by 19% in the elderly who engaged in physical activities ≥3 times weekly with at least 30 minutes per activity. The odds of depression also reduced by 13% and 22% among elderly who engaged in ≥1 social activity and religious activity weekly, respectively. Our community-based 3-year longitudinal study suggested not only that each of the three types of activity is individually associated with a lower risk of depression, but also that the combination of two or three types of activity is associated with much lower risk of depression. The odds of depression reduced by 36% among elderly who engaged in two or three types of activity, compared with the elderly who did not participate in any type of physical, social, and religious activity.

The significance of the current study lies in the fact that it is the first comprehensive approach that has been developed, to the best of the authors’ knowledge, to evaluate the protective effects of the three variables i.e., physical, social, and religious activity participation. The protective effects of these variables have been demonstrated in this study. Development of methods for reducing the risk of depression in the elderly has thus far been a critical issue in the field of community mental health service. Many researchers individually proposed different psychosocial risk factors that were considered important from their own viewpoints, and also conducted relevant intervention studies [48,49].

In practice, however, mental health promotion for the elderly in the community seldom targets only a single risk factor, but usually targets various risk factors of similar characteristics simultaneously. Accordingly, the necessity of research pursuing concrete evidence on the correlation or combination effect of these activities, not just presumption, has continuously been cited. In the current study, we demonstrated that the combination of two or three types of activity further reduces the risk of depression. In addition, the insignificant or negligible correlation between these three activities, which are thought to be similar and interactive, is considered an important and meaningful finding for developing a mental health promotion or intervention study. Few preceding studies have suggested similar results [26,31,50]. Therefore, future mental health promotion or intervention studies on physical, social, and religious activity need to be undertaken and emphasized independently, to strengthen their protective effect.

Unfortunately, the present research did not involve an intervention study design and therefore could not provide a completely satisfactory answer. In addition, consideration of diverse variables together made it difficult to adequately explain the mechanism of the protective effects. Nevertheless, our evidence-based approach is very specific, intuitive, realizable, and relatively easy e.g., *“You can reduce the risk of depression by taking part in three sessions of physical activity weekly*, *each session lasting more than 30 minutes*, *one session of social activity weekly*, *and one session of religious activity weekly*.*”* Intervention studies would be necessary in the future for further experimental evidence. In addition, this cut-off point should not be considered an absolute value, because other factors such as the sociocultural characteristics would also have to be considered.

The study had multiple strengths. First, study subjects were from a relatively large nationwide longitudinal community-based survey in Korea. Second, adjustment was not restricted to definite or potential demographic risk factors for depression, but also to clinically important risk factors such as smoking, drinking, comorbidity, disability, cognitive decline, and baseline depressive symptoms. Finally, the present study defined intuitive and easy-to-measure independent variables and achieved positive results. The study therefore presented further evidence for planning an intervention study or public campaigns related to lifestyle factors.

Despite its strengths, our study had some limitations. First, the assessment of depression was based on a screening tool rather than a definite diagnostic measure or clinical interview by a doctor. Second, owing to the observational design of the study, unmeasured confounders may have resulted in biased outcome. Third, the physical, social, and religious participation of the study participants could not be fully confirmed through an interview, but depended on their responses to a survey. Such limitations could also lead to recall bias. Therefore, to minimize recall bias, subjects who showed cognitive decline were excluded. Fourth, the representativeness and generalizability of our study were limited. This limitation could result from selection bias, which was probably a consequence of the initial recruitment process conducted via telephone, and the application of operational inclusion and exclusion criteria for the LPOPS. In addition, insufficient consideration of regional social context, which cannot be covered by single level regression analysis, could also limit the representativeness and generalizability of this study. Fifth, insufficient information about the quality and quantity of physical activity from the self-reported non-standardized measure is another limitation that must be acknowledged. Future work is needed to attain a more specific, validated measure, such as a metabolic equivalent [51]. Sixth, the study addressed only the quantitative aspects, and not functional or qualitative aspects, of the three types of activity. Considering the variability in physical, social, and religious activities, future research requires focusing on specific details and qualitative aspects of the aforementioned lifestyles. Finally, group classification in the present study made it difficult to provide a sufficient explanation or mechanism for the combinatorial effect of activities on depression.

## Conclusion

Participation in physical, social, and religious activity was associated with decreased risk of depression in the elderly. In addition, the risk of depression was much lower in the elderly who participated in two or three of the above-mentioned types of activity than that in the elderly who did not.

## References

[1] Arean PA, Reynolds CF 3rd. The impact of psychosocial factors on late-life depression. Biol Psychiatry. 2005;58: 277–282. 16102545 10.1016/j.biopsych.2005.03.037

[2] Bruce ML. Psychosocial risk factors for depressive disorders in late life. Biol Psychiatry. 2002;52: 175–184. 12182924 10.1016/s0006-3223(02)01410-5

[3] Forsman AK, Schierenbeck I, Wahlbeck K. Psychosocial interventions for the prevention of depression in older adults: systematic review and meta-analysis. J Aging Health. 2011;23: 387–416. 10.1177/0898264310378041 20935250

[4] Forsman AK, Nordmyr J, Wahlbeck K. Psychosocial interventions for the promotion of mental health and the prevention of depression among older adults. Health Promot Int. 2011;26 Suppl 1: i85–107. 10.1093/heapro/dar074 22079938

[5] Sarris J, O'Neil A, Coulson CE, Schweitzer I, Berk M. Lifestyle medicine for depression. BMC Psychiatry. 2014;14: 107 10.1186/1471-244X-14-107 24721040 PMC3998225

[6] Lucas M, Mekary R, Pan A, Mirzaei F, O'Reilly EJ, Willett WC, et al Relation between clinical depression risk and physical activity and time spent watching television in older women: a 10-year prospective follow-up study. Am J Epidemiol. 2011;174: 1017–1027. 10.1093/aje/kwr218 21984659 PMC3243936

[7] Pasco JA, Williams LJ, Jacka FN, Henry MJ, Coulson CE, Brennan SL, et al Habitual physical activity and the risk for depressive and anxiety disorders among older men and women. Int Psychogeriatr. 2011;23: 292–298. 10.1017/S1041610210001833 20863424

[8] Shankar A, McMunn A, Banks J, Steptoe A. Loneliness, social isolation, and behavioral and biological health indicators in older adults. Health Psychol. 2011;30: 377–385. 10.1037/a0022826 21534675

[9] Hayward RD, Owen AD, Koenig HG, Steffens DC, Payne ME. Religion and the presence and severity of depression in older adults. Am J Geriatr Psychiatry. 2012;20: 188–192. 10.1097/JGP.0b013e31822ccd51 22273738 PMC3266521

[10] Bridle C, Spanjers K, Patel S, Atherton NM, Lamb SE. Effect of exercise on depression severity in older people: systematic review and meta-analysis of randomised controlled trials. Br J Psychiatry. 2012;201: 180–185. 10.1192/bjp.bp.111.095174 22945926

[11] Park JE, Lee JY, Kim BS, Kim KW, Chae SH, Cho MJ. Above-moderate physical activity reduces both incident and persistent late-life depression in rural Koreans. Int J Geriatr Psychiatry. 2014. 10.1002/gps.4244 25503946

[12] Erickson KI, Gildengers AG, Butters MA. Physical activity and brain plasticity in late adulthood. Dialogues Clin Neurosci. 2013;15: 99–108. 23576893 10.31887/DCNS.2013.15.1/kerickson PMC3622473

[13] Meeusen R, De Meirleir K. Exercise and brain neurotransmission. Sports Med. 1995;20: 160–188. 8571000 10.2165/00007256-199520030-00004

[14] Wassink-Vossen S, Collard RM, Oude Voshaar RC, Comijs HC, de Vocht HM, Naarding P. Physical (in)activity and depression in older people. J Affect Disord. 2014;161: 65–72. 10.1016/j.jad.2014.03.001 24751309

[15] World Health Organization. Active ageing: a policy framework. Madrid, Spain. Available: http://whqlibdoc.who.int/hq/2002/WHO_NMH_NPH_02.8.pdf.

[16] Cruwys T, Dingle GA, Haslam C, Haslam SA, Jetten J, Morton TA. Social group memberships protect against future depression, alleviate depression symptoms and prevent depression relapse. Soc Sci Med. 2013;98: 179–186. 10.1016/j.socscimed.2013.09.013 24331897

[17] Chiao C, Weng LJ, Botticello AL. Social participation reduces depressive symptoms among older adults: an 18-year longitudinal analysis in Taiwan. BMC Public Health. 2011;11: 292 10.1186/1471-2458-11-292 21569285 PMC3103460

[18] Dickens AP, Richards SH, Greaves CJ, Campbell JL. Interventions targeting social isolation in older people: a systematic review. BMC Public Health. 2011;11: 647 10.1186/1471-2458-11-647 21843337 PMC3170621

[19] Sugihara Y, Sugisawa H, Shibata H, Harada K. Productive roles, gender, and depressive symptoms: evidence from a national longitudinal study of late-middle-aged Japanese. J Gerontol B Psychol Sci Soc Sci. 2008;63: P227–P234. 18689764 10.1093/geronb/63.4.p227

[20] Uebelacker LA, Eaton CB, Weisberg R, Sands M, Williams C, Calhoun D, et al Social support and physical activity as moderators of life stress in predicting baseline depression and change in depression over time in the Women's Health Initiative. Soc Psychiatry Psychiatr Epidemiol. 2013;48: 1971–1982. 10.1007/s00127-013-0693-z 23644722 PMC3796164

[21] Teo AR, Choi H, Valenstein M. Social relationships and depression: ten-year follow-up from a nationally representative study. PLoS One. 2013;8: e62396 10.1371/journal.pone.0062396 23646128 PMC3640036

[22] Chen H, Cheal K, McDonel Herr EC, Zubritsky C, Levkoff SE. Religious participation as a predictor of mental health status and treatment outcomes in older persons. Int J Geriatr Psychiatry. 2007;22: 144–153. 17245799 10.1002/gps.1704

[23] Braam AW, Hein E, Deeg DJ, Twisk JW, Beekman AT, Van Tilburg W. Religious involvement and 6-year course of depressive symptoms in older Dutch citizens: results from the Longitudinal Aging Study Amsterdam. J Aging Health. 2004;16: 467–489. 15271266 10.1177/0898264304265765

[24] Hahn CY, Yang MS, Yang MJ, Shih CH, Lo HY. Religious attendance and depressive symptoms among community dwelling elderly in Taiwan. Int J Geriatr Psychiatry. 2004;19: 1148–1154. 15526305 10.1002/gps.1204

[25] Koenig HG. Religion and depression in older medical inpatients. Am J Geriatr Psychiatry. 2007;15: 282–291. 17384313 10.1097/01.JGP.0000246875.93674.0c

[26] Koenig HG, Hays JC, George LK, Blazer DG, Larson DB, Landerman LR. Modeling the cross-sectional relationships between religion, physical health, social support, and depressive symptoms. Am J Geriatr Psychiatry. 1997;5: 131–144. 9106377

[27] Zou J, Huang Y, Maldonado L, Kasen S, Cohen P, Chen H. The efficacy of religious service attendance in reducing depressive symptoms. Soc Psychiatry Psychiatr Epidemiol. 2014;49: 911–918. 10.1007/s00127-013-0785-9 24178134

[28] Miller L, Bansal R, Wickramaratne P, Hao X, Tenke CE, Weissman MM, et al Neuroanatomical correlates of religiosity and spirituality: a study in adults at high and low familial risk for depression. JAMA Psychiatry. 2014;71: 128–135. 10.1001/jamapsychiatry.2013.3067 24369341 PMC3921896

[29] Manning LK. Navigating hardships in old age: exploring the relationship between spirituality and resilience in later life. Qual Health Res. 2013;23: 568–575. 10.1177/1049732312471730 23282796 PMC3578989

[30] Krause N. Religious meaning and subjective well-being in late life. J Gerontol B Psychol Sci Soc Sci. 2003;58: S160–170. 12730317 10.1093/geronb/58.3.s160

[31] Correa AA, Moreira-Almeida A, Menezes PR, Vallada H, Scazufca M. Investigating the role played by social support in the association between religiosity and mental health in low income older adults: results from the Sao Paulo Ageing & Health Study (SPAH). Rev Bras Psiquiatr. 2011;33: 157–164. 21829909 10.1590/s1516-44462011000200011

[32] Schwarzbach M, Luppa M, Forstmeier S, Konig HH, Riedel-Heller SG. Social relations and depression in late life-a systematic review. Int J Geriatr Psychiatry. 2014;29: 1–21. 10.1002/gps.3971 23720299

[33] Son J, Lin N, George LK. Cross-national comparison of social support structures between Taiwan and the United States. J Health Soc Behav. 2008;49: 104–118. 18418988 10.1177/002214650804900108

[34] Miller L, Wickramaratne P, Gameroff MJ, Sage M, Tenke CE, Weissman MM. Religiosity and major depression in adults at high risk: a ten-year prospective study. Am J Psychiatry. 2012;169: 89–94. 10.1176/appi.ajp.2011.10121823 21865527 PMC3547523

[35] Razafsha M, Behforuzi H, Azari H, Zhang Z, Wang KK, Kobeissy FH, et al Qualitative versus quantitative methods in psychiatric research. Methods Mol Biol. 2012;829: 49–62. 10.1007/978-1-61779-458-2_3 22231806

[36] Lee Y, Kim J, Han ES, Ryu M, Cho Y, Chae S. Frailty and body mass index as predictors of 3-year mortality in older adults living in the community. Gerontology. 2014;60: 475–482. 10.1159/000362330 24993678

[37] Han ES, Lee Y, Kim J. Association of cognitive impairment with frailty in community-dwelling older adults. Int Psychogeriatr. 2014;26: 155–163. 10.1017/S1041610213001841 24153029

[38] Yesavage JA, Sheikh JI. 9/Geriatric Depression Scale (GDS). Clin Gerontol. 1986;5: 165–173.

[39] Bae JN, Cho MJ. Development of the Korean version of the Geriatric Depression Scale and its short form among elderly psychiatric patients. J Psychosom Res. 2004;57: 297–305. 15507257 10.1016/j.jpsychores.2004.01.004

[40] Lee SC, Kim WH, Chang SM, Kim BS, Lee DW, Bae JN, et al The Use of the Korean Version of Short Form Geriatric Depression Scale (SGDS-K)in the Community Dwelling Elderly in Korea. J Korean Geriatr Psychiatry. 2013;17: 36–42.

[41] Mura G, Carta MG. Physical activity in depressed elderly. A systematic review. Clin Pract Epidemiol Ment Health. 2013;9: 125–135. 10.2174/1745017901309010125 24009640 PMC3758953

[42] Guerra M, Ferri CP, Sosa AL, Salas A, Gaona C, Gonzales V, et al Late-life depression in Peru, Mexico and Venezuela: the 10/66 population-based study. Br J Psychiatry. 2009;195: 510–515. 10.1192/bjp.bp.109.064055 19949200 PMC2915389

[43] Harris T, Cook DG, Victor C, Rink E, Mann AH, Shah S, et al Predictors of depressive symptoms in older people—a survey of two general practice populations. Age Ageing. 2003;32: 510–518. 12958000 10.1093/ageing/afg087

[44] Won CW, Yang KY, Rho YG, Kim SY, Lee EJ, Yoon JL, et al The Development of Korean Activities of Daily Living(K-ADL) and Korean Instrumental Activities of Daily Living(K-IADL) Scale. J Korean Geriatr Soc. 2002;6: 107–120.

[45] Won CW, Rho YG, SunWoo D, Lee YS. The Validity and Reliability of Korean Instrumental Activities of Daily Living(K-IADL) Scale. J Korean Geriatr Soc. 2002;6: 273–280.

[46] Lee DY, Lee KU, Lee JH, Kim KW, Jhoo JH, Youn JC, et al A Normative Study of the Mini-Mental State Examination in the Korean Elderly. J Korean Neuropsychiatr Assoc. 2002;41: 508–525.

[47] Lee KS, Cheong HK, Oh BH, Hong CH. Comparison of the Validity of Screening Tests for Dementia and Mild Cognitive Impairment of the Elderly in a Community: K-MMSE, MMSE-K, MMSE-KC, and K-HDS. J Korean Neuropsychiatr Assoc. 2009;48: 61–69.

[48] Oyama H, Ono Y, Watanabe N, Tanaka E, Kudoh S, Sakashita T, et al Local community intervention through depression screening and group activity for elderly suicide prevention. Psychiatry Clin Neurosci. 2006;60: 110–114. 16472368 10.1111/j.1440-1819.2006.01468.x

[49] Maki Y, Ura C, Yamaguchi T, Takahashi R, Yamaguchi H. Intervention using a community-based walking program is effective for elderly adults with depressive tendencies. J Am Geriatr Soc. 2012;60: 1590–1591. 10.1111/j.1532-5415.2012.04091.x 22889030

[50] Kim KH, Sobal J. Religion, social support, fat intake and physical activity. Public Health Nutr. 2004;7: 773–781. 15369616 10.1079/phn2004601

[51] Ainsworth BE, Haskell WL, Herrmann SD, Meckes N, Bassett DR Jr, Tudor-Locke C, et al 2011 Compendium of Physical Activities: a second update of codes and MET values. Med Sci Sports Exerc. 2011;43: 1575–1581. 21681120 10.1249/MSS.0b013e31821ece12

